# The Patient and Family Narratives seminars at the University of Saskatchewan connect health professions students with patient experiences

**DOI:** 10.36834/cmej.68906

**Published:** 2020-09-23

**Authors:** Marcel F. D’Eon, Jelyssa Luc

**Affiliations:** 1 University of Saskatchewan, Saskatchewan, Canada

## Implication Statement

The Patient and Family Narratives (PFN) seminars make patient perspectives part of the education of students from healthcare professions. These sessions consist of a story shared by a patient, interdisciplinary small group discussions, and a question and answer period. Based on the findings of our evaluation, we will modify the orientation exercise and small group sessions. The simple design of these seminars makes their implementation suitable for other institutions as a way of sharing patient stories in an interprofessional setting. However, the success of a PFN program may depend on a lack of authentic patient contact in the pre-existing curriculum.

## Déclaration des répercussions

Les séminaires sur les récits des patients et de leur famille (PFN) intègrent la perspective du patient dans l’éducation des étudiants des professions de la santé. Ces séances comportentune histoire partagée par un patient, des discussions interdisciplinaires en petits groupes et une période de questions et réponses. Selon les résultats de notre évaluation, nous modifierons l’exercice d’accueil et les sessions en petits groupes. Le devis simple de ces séminaires rend leur mise en œuvre appropriée pour d’autres établissements comme une manière de partager les histoires des patients dans un cadre de travail interprofessionnel. Toutefois, le succès de ce programme peut dépendre du manque de contact authentique avec le patient dans le cursus préexistant.

The patient’s experience of their illness^1^ along with their unique social, emotional, and spiritual situation are important considerations in providing effective and compassionate health care.^2^^-^^4^ The Patient and Family Narratives (PFN) seminars at the University of Saskatchewan were created to provide a venue for students from health professions programs to experience patients’ stories of their journeys through illness and treatment. Beginning in 2010, an interprofessional team from the University of Saskatchewan, Saskatchewan Polytechnic, and the Saskatchewan Health Authority has organized several PFN sessions annually providing up to 500 individual student experiences per year.

Over the past 10 years, most students attended only a few of the three to seven sessions offered and not always the first session. We needed a way to communicate the purpose and agenda of the PFN sessions for students who may join us throughout the year without taking up time during the sessions and repeating introductory information for those who have already attended one or more sessions. We decided that each student, prior to attending their first session, be required to complete an orientation exercise. This consisted of emailing a brief summary of the purpose of the PFN seminars and a description of what they hoped to learn to the PFN organizers.

There were two main parts to the PFN sessions. The core of the PFN sessions consisted of a patient or patients, often accompanied by a caregiver and/or family member, sharing parts of their personal illness story and encounters with the health care system. Patients were given broad guidelines to support the presentation of their stories. After the patient story, in small interprofessional groups, students discussed questions provided by the patient presenter and/or the faculty facilitators. Students then reconvened in the large group to discuss the findings of their small groups and often to ask questions of the patient presenter.

We compiled and analyzed hundreds of student comments written following each of the seven PFN seminars offered during the 2018-2019 academic year and other student comments collected from previous years. We found three themes: overall satisfaction with the seminars, an extraordinary appreciation for the patient stories, and moderately positive experiences with the small group interprofessional discussions and the orientation exercise.

To investigate the strength of the student sentiments, we developed a survey that we distributed in July 2019 to the 2018-2019 PFN student attendees. The survey asked participants to rate several features of the PFN utilizing a 6-point scale with options ranging from “strongly dissatisfied” to “strongly satisfied.” Both the analysis of the comments collected during the year and the survey were granted exemption from ethics review by the University of Saskatchewan Behavioral Research Ethics Board since this was designated a program evaluation.

In total 50 students from four programs (response rate of 25%) completed our survey representing over 150 individual experiences (see Table 1). The satisfaction ratings were out of 6.0. The mean for the PFN seminars overall was 4.3 (mode 4.0) while the mean for the patient stories alone was 4.9 (mode 5.0). The small group discussions and orientation were not rated as highly: means of 3.3 and 3.7 (mode 4.0 and 4.0) respectively.

**Table 1:** Survey results

Survey Questions (max=6)	Mean (Mode)
Number of PFN sessions attended	3.2 (4.0)
Overall satisfaction with PFN	4.3 (4.0)
Satisfaction with patient stories	4.9 (5.0)
Satisfaction with group discussion	3.3 (4.0)
Satisfaction with orientation exercise	3.7 (4.0)

The PFN seminars have been very successful judging by the large numbers of students who have participated and how highly students rated the patient stories. Furthermore, recall bias in the results presented is unavoidable due to the nature of the study. Moving forward, we are offering PFN sessions in 2019-2020 but have improved the structure of the small interprofessional groups and the orientation. We believe that the PFN seminars have enhanced the education of health professions students by connecting them with compelling patient stories.
